# Cold intolerance and associated factors: a population study

**DOI:** 10.1038/s41598-022-21842-9

**Published:** 2022-10-27

**Authors:** Alireza Khabbazi, Rojin Farzaneh, Maryam Mahmoudi, Mohammad Shahi, Amirreza Jabbaripour Sarmadian, Elaheh Babapour, Leila Alizadeh, Raha Khabbazi, Masoud Nouri-Vaskeh

**Affiliations:** 1 Connective Tissue Diseases Research Center, Tabriz University of Medical Sciences, Imamreza Hospital, Flat 1, P.O Box 5166614756, Tabriz, Iran; 2 Liver and Gastrointestinal Diseases Research Center, Tabriz University of Medical Sciences, Tabriz, Iran

**Keywords:** Physiology, Climate sciences, Environmental sciences, Rheumatology

## Abstract

Cold intolerance has been defined as a set of symptoms including pain, tingling, numbness, chills, stiffness, weakness, swelling or skin color changes on exposure to cold. Cold intolerance may have a profound effect on health-related quality of life. In this cross-sectional study, we investigated primarily the prevalence of cold intolerance and secondly associated factors in the general population of Tabriz. Simple random sampling of individuals aged ≥ 18 was performed from the population covered by Emamieh health center under the supervision of Tabriz University of Medical Sciences**.** A telephone interview was conducted with the participants by the general physician of that center. In participants with a positive response to each of two questions “I am oversensitive to cold” and “I experience pain or discomfort when exposed to cold” a Cold Intolerance Symptom Severity (CISS) questionnaire was filled. We used a cut off value 50 for defining cold intolerance. Of the 353 person who received telephone calls, 322 answered questions. Cold related symptoms and cold intolerance were reported in 144 (44.7%) and 38 (11.1%) persons, respectively. Cold intolerance was significantly more common in females and people with comorbidities. Cold intolerance led to a decrease in quality of job in 27 (8.4%) and a change in job in 6 (1.9%) persons. In conclusion, cold intolerance is a common problem in the general population of Tabriz.

## Introduction

Cold intolerance or cold sensitivity has been defined as a set of symptoms including pain, tingling, numbness, chills, stiffness, weakness, swelling or skin color changes on exposure to cold. Neurovascular, humoral and endocrine factors play role in the pathogenesis of cold intolerance^1^. Recently, the role of genetic factors in determining the intensity of cold induced pain in different people has been considered^2^. Cold intolerance has been reported in many conditions including upper extremity injuries and surgeries, fibromyalgia, anemia, hypothyroidism, atherosclerosis, Raynaud’s disease, diabetes, low body weight, vitamin B12 deficiency, Fabry disease, side effects of medications, hypothalamus diseases, paroxysmal cold hemoglobinuria and Waldenstrom’s macroglobulinemia^1,3,4^. Carlsson et al. reported cold intolerance in 45% of person with history of traumatic hand injuries^5^. Novak et al. reported cold intolerance in 30% of patients with hand related traumatic and non-traumatic pathologies^6^. Klocker et al. reported cold intolerance in 41% of patients underwent repair of upper limb arterial injuries^7^.

Cold intolerance may have a profound effect on health-related quality of life^5^. Cold intolerance is one of the main reasons of disability after hand injuries or surgeries^7–11^. Despite many studies that reported the frequency of cold intolerance after upper limb surgeries, the literature on the prevalence of cold intolerance in the general population is scarce. Information about the prevalence of cold intolerance in the general population can be used to promote public health and assess the frequency of this health problem in various medical conditions. In this cross-sectional study, we investigated primarily the prevalence of cold intolerance and secondly associated factors in the general population of Tabriz.

## Methods

This cross-sectional study was conducted from September 5, 2021 to January 18, 2022 in Tabriz. The city of Tabriz is the largest city in northwestern Iran with a population of more than 1.5 million people. Tabriz has a Continental climate with regular seasons bordering cold semi-arid climate. Simple random sampling of individuals aged ≥ 18 was performed from the population covered by Emamieh health center under the supervision of Tabriz University of Medical Sciences. Comparison of age distribution, sex and educational level of participants with Tabriz population aged ≥ 18 showed that study population is reliably representative of Tabriz population (Table 1)^12^. A telephone interview was conducted with the participants by the trained general physician (GP) of that center. Due to the fact that the population covered by this health center was closely followed, the participation rate was more than 90%. A questionnaire contained questions about age, sex, weight, height, job, smoking, family history of cold intolerance, current medications, underlying diseases, history of frostbite, history of hand injuries and surgeries, cold intolerance symptoms and effect of cold intolerance on job was filled out during interview and completed and rechecked by their medical records. In participants with a positive response to each of the two questions “I am oversensitive to cold” and “I experience pain or discomfort when exposed to cold” a Cold Intolerance Symptom Severity (CISS) questionnaire was filled. The CISS score evaluates 6 domains with various subsets of questions^13^. Scores range from 4 to 100, and higher scores indicate worse symptoms^13^. We used a cut off value 50 for defining cold intolerance^14^. The English version of CISS was translated into Farsi language by two independent bilingual expert translators. Discrepancies between translators were discussed and resolved. Then backward translation was done by two independent bilingual translators. They were blinded to the concept of investigation. After that an expert committee reviewed all versions of the translations and resolved discrepancies. We tested the prefinal version on 50 independent respondents and after interviewing the respondents, ensured that the translated items retained the same meaning as the original items. We translated the CISS questionnaire according to recommended methodology for translating a questionnaire^15^. Due to the presence of illiterate and low-educated people in the investigated samples, the questionnaire was completed during a telephone interview by the GP of the center. Severity of cold sensitivity in exposure to cold weather was assessed using a 100 mm visual analogue scale (VAS). A decreased in quality of job was defined as a decrease in wellbeing or satisfaction or income. The study was conducted in accordance with the Declaration of Helsinki and its protocol was approved by the Ethics Committee of Tabriz University of Medical Sciences. Informed consent has been obtained from all participants. Then, we performed a pilot study on 30 participants. Cold intolerance was detected in 4 cases. Considering *P* = 0.133, d = 0.038 and type 1 error (0.05), the sample size was estimated to be 307 people $$\left( {{\text{n}} = {\text{z }}{\raise0.7ex\hbox{${\upalpha }$} \!\mathord{\left/ {\vphantom {{\upalpha } 2}}\right.\kern-\nulldelimiterspace} \!\lower0.7ex\hbox{$2$}}{ }\frac{{{\text{p}}\left( {1 - {\text{p}}} \right)}}{{{\text{d}}^{2} }}} \right)$$.

**Table 1:** Comparison of studied population and Tabriz population characteristics.

Parameters	Tabriz (*n* = 1,558,693)							Study population (*n* = 322)						
	18–29 (%)	30–39 (%)	40–49 (%)	50–59 (%)	60–69 (%)	> 70 (%)	Total (%)	18–29 (%)	30–39 (%)	40–49 (%)	50–59 (%)	60–69 (%)	≥ 70 (%)	Total (%)
**Gender**														
Female	22.1	27.5	20.1	14.9	9.0	6.4	100	23.8	26.8	20.7	13.4	9.8	5.5	100
Male	22.8	27.8	19.7	14.8	8.7	6.3	100	23.4	25.9	19.6	15.2	10.1	5.7	100
**Education level**														
Illiterate	1.7	3.4	10.5	24.6	39.8	62.9	10.1	1.3	4.0	4.6	7.8	9.0	60.0	12.1
Primary	5.9	11.4	22.5	24.8	27.1	22.2	23.3	2.6	17.3	47.7	39.2	28.9	33.3	25.5
Highschool	48.2	52.5	45.1	36.1	21.7	9.0	43.9	70.5	44.0	29.2	33.3	5.3	6.7	37.9
University	44.2	32.7	21.9	14.5	11.4	5.9	22.6	25.6	34.7	18.5	19.6	15.8	0	24.5

*n* number.

### Statistical analysis

Statistical analyses were conducted using the SPSS statistical package (SPSS Inc., version 16). The normal distribution of data was assessed using the Kolmogorov–Smirnov test. Qualitative and quantitative variables were displayed as numbers (percentages) and means ± standard deviation (SD), respectively. Comparisons between people with and without cold intolerance were made by chi-squared test. To reduce the chances of type I errors, the *p*-values obtained from the chi-squared test corrected using the Bonferroni method. *P*-values less than 0.00625 were considered as statistically significant.

## Results

Of the 353 person who received telephone calls, 322 answered questions. Demographic characteristics of participants were shown in Table 2. Cold-related symptoms were reported in 144 (44.7%) persons. Pain was the most common symptom when exposed to cold. Pain severity was 6.53 ± 2.47. Frequency of symptoms in exposure to cold were shown in Table 1. The mean CISS in the 144 subjects measured was 36.9 ± 18.6. Cold intolerance according CISS ≥ 50 was reported in 38 (11.1%) patients. Twenty-seven (8.4%) participants had a history of upper limb trauma or surgery or diseases and 18 (66.7%) of them had neuropathic pain. The frequency of cold intolerance in participants with upper limb conditions with and without and neuropathic pain was 55.6 and 33.3 percent, respectively. The difference was significant (P = 0.038). Cold intolerance was started during childhood or adolescence in 19 (50.0%) patients. In the other cases, it started at the ages of 18–30, 30–50 and ≥ 50 in 6 (15.7%), 9 (23.7%) and 9 (23.7%) cases, respectively. Cold intolerance led to a decrease in quality of job in 27 (8.4%) and a change in job in 6 (1.9%) persons.

**Table 2:** Demographic and health characteristics of participants (*n* = 322).

Parameters	*n* (%)
**Female (%)**	164 (50.9)
**Age**	
18–29 (%)	76 (23.6)
30–39 (%)	85 (26.4)
40–49 (%)	65 (20.2)
50–59 (%)	46 (14.3)
60–69 (%)	32 (9.9)
≥ 70 (%)	18 (5.6)
**BMI (mean ± SD**)	26.2 ± 4.2
**Smoker (%)**	32 (9.9)
**Education**	
Illiterate (%)	39 (12.1)
Primary school (%)	82 (25.5)
Highschool (%)	122 (37.9)
University (%)	79 (24.5)
**Comorbidities (%)**	88 (27.3)
Hypertension (%)	35 (10.9)
Diabetes (%)	30 (9.3)
Fibromyalgia (%)	21 (6.5)
Hyperlipidemia (%)	17 (5.3)
Thyroid disorders	16 (5.0)
Anemia (%)	6 (1.9)
Upper limb trauma (%)	13 (4.0)
Upper limb diseases (%)	12 (3.7)
Upper limb surgery (%)	12 (3.7)
Peripheral vascular diseases (%)	5 (1.6)
Rheumatic diseases (%)	4 (1.2)
**Cold related symptoms (%)**	144 (44.7)
Pain (%)	78 (54.2)
Numbness (%)	39 (27.1)
Shivering (%)	33 (22.9)
Skin color change (%)	19 (13.2)
Weakness (%)	14 (9.7)
Stiffness (%)	4 (2.8)
Swelling (%)	4 (2.8)
**Cold intolerance (%)**	38 (11.8)

*n* number, *BMI* body mass index, *SD* standard deviation.

*Upper limb trauma* any injury to arm, or to the fingers.

*Upper limb diseases* problems with the soft tissues, muscles, tendons and ligaments, circulation and nerve supply to the limb that cause aches, pains, tension involving any part of the arm from fingers to shoulder or the neck.

Demographic and clinical characteristics of participants with and without cold intolerance were shown in Table 3. After applying the Bonferroni correction, cold intolerance was significantly more common in females and people with comorbidities (Table 3).

**Table 3:** Frequency of cold intolerance in different groups.

Parameters	CISS ≥ 50 (*N* = 38)	CISS < 50 (*N* = 284)	*P*-value
**Age**			0.522
18–29 (%)	7 (18.4)	71 (25.0)	
30–39 (%)	7 (18.4)	68 (23.9)	
40–49 (%)	7 (18.4)	58 (20.4)	
50–59 (%)	10 (26.3)	41 (14.4)	
≥ 60 (%)	7 (18.4)	46 (16.2)	
**Female (%)**	27 (71.1)	137 (48.2)	0.006*
**Obesity (%)**	7 (18.9)	49 (17.3)	–
**Smoking (%)**	3 (7.9)	29 (10.2)	–
**Familial history of cold intolerance (%)**	14 (36.8)	59 (20.8)	0.021
**Comorbidities**	42 (43.3)	46 (20.4)	0.001*
Hypertension (%)	8 (21.1)	27 (9.5)	–
Diabetes (%)	7 (18.4)	23 (8.1)	–
Fibromyalgia (%)	11 (28.9)	10 (3.5)	–
Anemia (%)	4 (10.5)	2 (0.7)	–
Hypothyroidism (%)	3 (7.9)	13 (4.6)	–
Upper limb trauma (%)	4 (10.5)	9 (3.2)	–
Upper limb disease (%)	7 (18.4)	5 (1.8)	–
Upper limb surgery (%)	5 (13.2)	7 (2.5)	–
Peripheral vascular diseases (%)	1 (2.6)	4 (1.4)	–
Hypothalamic disorders (%)	0	0	–
Rheumatic diseases (%)	0	4 (1.4)	–
**Medications**			
Antihypertensive agents (%)	6 (15.8)	27 (9.5)	–
Beta blockers (%)	2 (5.3)	14 (4.9)	–
Alfa blockers (%)	0	1 (0.4)	–
Levothyroxine (%)	4 (10.5)	14 (4.9)	–
Statins (%)	4 (10.5)	13 (4.6)	–
Psychoactive medications (%)	4 (10.6)	17 (6.0)	–
Calcium blockers (%)	0	4 (1.4)	–

*After Bonferroni correction, they remained at significant levels.

*CISS* Cold Intolerance Symptom Severity.

## Discussion

In our study cold-related symptoms and cold intolerance were reported in 44.7 and 11.1 percent of studied population. The demographic characteristics of studied population shows that it can be considered as a representative of the general population of Tabriz^12^. Despite numerous studies on the frequency of cold intolerance in patients with upper limb surgeries and injuries, there are few reports on the frequency of cold intolerance in the general population. Carlsson et al. reported frequency of self-reported cold intolerance in 5% of the normal population in Sweden^5^. In another study Stjernbrandt et al. reported cold intolerance in 9.7% of men and 14.4% of women in Sweden^16^. They found a positive correlation between cumulative cold exposure and cold intolerance^16^. Näyhä et al. reported cold-related symptoms in 45% of population in Finland^17^. However, it should be noticed that they did not report the prevalence of cold intolerance based on the CISS^17^. In a recent report prevalence of cold-related symptoms among Thai poultry industry workers was 76.1%^18^.

Finger pain and numbness and shivering were the most common cold related symptoms in our studied cases. In Raatikka et al. report the most common symptoms were pain, shortness of breath and increased excretion of mucus^19^. However, in Auttanate et al. report the most common cold-related symptoms were respiratory symptoms^18^.

In our studied cases cold intolerance was more common in females, people with comorbidities and familial history of cold intolerance. Although after Bonferroni correction the higher frequency of cold intolerance reached a significant level only in females and people with comorbidities. These findings were in line with the findings of Mäkinen et al. who reported more susceptibility to cold in people with comorbidities like cardiovascular diseases, cerebrovascular diseases, diabetes and respiratory diseases^20^. Serlani et al. have reported more cold-induced pain in the face of the same cold intensity in women compared to men^21^. Näyhä et al. reported that cold-related symptoms in cardiovascular and respiratory systems were more common among women than men^17^. Stjernbrandt et al. reported that frostbite affecting the hands (OR 10.3) is the strongest risk factor for cold intolerance^1^. In addition, upper extremity nerve injury (OR 2.0), and having rheumatic disease (OR 3.1), migraines (OR 2.4) and vascular disease (OR 1.9) were associated with cold sensitivity^1^. In present study, the frequency of cold intolerance was higher in participants with a history of surgery or trauma or diseases of the upper limbs who had neuropathic pain than in participants with no neuropathic pain. These results were consistent with the report of Magistroni et al. who reported a higher CISS score in participants with history of upper limb injuries^22^. We could not find associations between cold intolerance and age, body mass index (BMI). Collins et al. in a study on 50 patients with upper-extremity peripheral nerve injuries did not report association between cold sensitivity with age and smoking^23^. Similarly, Ruijs et al. did not report association between cold sensitivity with age in general population^13^. However, there were differences between our results and some previous studies. In the report of Stjernbrandt et al. contrary to our results, a BMI ≥ 25 was a negative risk factor for cold intolerance^1^. In a study on 198 patients with traumatic and non-traumatic hand pathologies, there was no difference in the prevalence of cold-induced symptoms between men and women^6^. In another study on patients with arterial repair in upper extremity injuries no association was reported between sex and age with cold intolerance^7^.

In our studied population cold intolerance led to a decrease in quality of job in 8.4% and a change in job in 1.9%. In Collin et al. report on patients who were followed after upper-extremity peripheral nerve injuries no association was observed between the presence or absence of cold intolerance and job change^15^. Carlsson et al. reported disturbance in job in 27% of patients with traumatic hand injuries or hand-arm vibration syndrome, and also higher CISS scores [66 (21–86)] in patients who changed jobs than those who remained in their previous jobs [45 (7–85)]^5^. In another report, cold-related job quality decline was more common among poultry industry workers who consumed alcohol weekly than non-drinkers^18^.

This study for the first time reported the frequency of cold intolerance in Iran. Population based design of the study and high rate of participation were the advantage of our study. Important limitations of the study were i) the relatively small sample size that did not provide a sufficient number of cases in various groups and subgroups, ii) use of CISS to diagnose cold intolerance and iii) risk of interviewer bias. The CISS was originally developed to measure cold intolerance in patients with upper limb surgeries and trauma and does not assess cold-related symptoms in other organs such as the respiratory and cardiovascular systems. In addition, we did not assess exposure time to cold and its effect on developing cold intolerance. Although the questions in the CISS are objective and the general practitioner who completed the CISS was trained, the possibility of interviewer bias should not be overlooked.

## Conclusion

Cold intolerance is a common problem in the general population. Cold intolerance is more common in females and people with comorbidities. However, a study with larger sample size is necessary for detecting independent risk factors.

## Data Availability

Data available on request. The data underlying this article will be shared on reasonable request to the corresponding author.
